# Effects of *Laccaria bicolor* on Gene Expression of *Populus trichocarpa* Root under Poplar Canker Stress

**DOI:** 10.3390/jof7121024

**Published:** 2021-11-29

**Authors:** Fengxin Dong, Yihan Wang, Ming Tang

**Affiliations:** 1 College of Forestry, Northwest A&F University, Xianyang 712100, China; 2018060258@nwsuaf.edu.cn (F.D.); 2018060256@nwafu.edu.cn (Y.W.); 2 State Key Laboratory of Conservation and Utilization of Subtropical Agro-Bioresources, Guangdong Laboratory for Lingnan Modern Agriculture, Guangdong Key Laboratory for Innovative Development and Utilization of Forest Plant Germplasm, College of Forestry and Landscape Architecture, South China Agricultural University, Guangzhou 510642, China

**Keywords:** *Botryosphaeria dothidea*, *Populus trichocarpa*, *Laccaria bicolor*, transcriptome analysis, disease resistance

## Abstract

Poplars can be harmed by poplar canker. Inoculation with mycorrhizal fungi can improve the resistance of poplars to canker, but the molecular mechanism is still unclear. In this study, an aseptic inoculation system of *L. bicolor*–*P. trichocarpa*–*B. dothidea* was constructed, and transcriptome analysis was performed to investigate regulation by *L. bicolor* of the expression of genes in the roots of *P. trichocarpa* during the onset of *B. dothidea* infection, and a total of 3022 differentially expressed genes (DEGs) were identified. Weighted correlation network analysis (WGCNA) was performed on these DEGs, and 661 genes’ expressions were considered to be affected by inoculation with *L. bicolor* and *B. dothidea*. Gene ontology (GO) and Kyoto Encyclopedia of Genes and Genomes (KEGG) enrichment analyses showed that these 661 DEGs were involved in multiple pathways such as signal transduction, reactive oxygen metabolism, and plant-pathogen interaction. Inoculation with *L. bicolor* changed the gene expression pattern of the roots, evidencing its involvement in the disease resistance response of *P. trichocarpa*. This research reveals the mechanism of *L. bicolor* in inducing resistance to canker of *P. trichocarpa* at the molecular level and provides a theoretical basis for the practical application of mycorrhizal fungi to improve plant disease resistance.

## 1. Introduction

Poplar canker, a disease caused by necrotrophic fungal pathogens, mainly damages the branches of poplars [1]. It is found worldwide, hindering the development of national forestry and causing economic losses to varying degrees [2,3,4,5]. At present, the prevention measures of poplar canker mainly include physical felling and chemical spraying, but these methods cause environmental pollution and economic losses. Therefore, a safe and effective method is needed to prevent or treat poplar canker.

Beneficial microorganisms present in soil have the potential to prevent plant diseases [6]. Mycorrhizal fungi are one kind of important beneficial symbiotic fungi found in soil. Ectomycorrhizal fungi (ECMF) [7] and arbuscular mycorrhizal fungi (AMF) [8] can establish a symbiotic relationship with *Populus* species. They not only enhance the absorption of nutrients and minerals by plants but also improve the ability of plants to resist disease, such as poplar canker [9,10,11,12,13,14]. Therefore, poplar canker can be controlled by inoculating poplars with mycorrhiza fungi. For example, *Xerocomus chrysenteron* has been used to control poplar canker in the field, and the control effect (((disease index of control groups—disease index of treatment groups)/disease index of control groups) × 100%) reached 54.5% [15].

The invasion of pathogenic fungi destroys cell membranes, causes membrane lipid peroxidation, produces reactive oxygen species (ROS) and malondialdehyde (MDA), and expands the damage range [16]. Plants can reduce these negative effects by regulating the activity of some defense enzymes, such as peroxidase (POD) and L-phenylalanine ammonia-lyase (PAL) [17]. The increase in POD activity can promote the oxidation of phenol to quinone, which is harmful to pathogenic fungi. PAL is one of the main enzymes of phenol metabolism, and it affects the synthesis of phenolic compounds [18,19]. Inoculation with *Boletus luridus* and *Glomus mosseae* has been shown to reduce the incidence of poplar canker; increase the activity of POD and PAL in the roots, stems, and leaves; and reduce the content of MDA [20]. Mycorrhizal fungi can change the enzyme activity in the stems and leaves of plants, which may be achieved by changing the expression of plant roots and then the gene expression of stems and leaves [21]. However, the effect of mycorrhizal fungi on host root gene expression under disease stress is limited [22,23].

In the process of the interaction between plants and pathogenic fungi, a series of signal transmissions occur in the plant to activate the plant’s defense system, including hormone signal transduction pathways and ROS signal transduction pathways [24,25,26,27,28]. Signal molecules participate in the connection between roots and stems. Mycorrhizal fungi participate in the disease-resistance process of stems and leaves and may rely on the transduction of signal molecules to change the activity of disease-resistant substances. Therefore, the regulatory role of mycorrhizal fungi in disease-resistant signal transduction pathways also requires detailed investigation.

Transcriptome sequencing analysis can reveal metabolic regulation mechanisms at the molecular level and has become an indispensable method for studying gene expression, RNA translation, and metabolism [29,30]. *B. dothidea* is one of the main pathogenic fungi of poplar canker in China. *Populus trichocarpa* and *Laccaria bicolor* are model organisms (representing poplars and mycorrhizal fungi, respectively). In this study, an aseptic inoculation system of *L. bicolor–P. trichocarpa–B. dothidea* was constructed and transcriptome analysis was performed to investigate the regulation of *L. bicolor* on the expression of genes in the roots of *P. trichocarpa* during the onset of *B. dothidea*. In this way, we can explore the gene expression patterns of mycorrhizal roots under disease stress.

## 2. Materials and Methods

### 2.1. Plant and Fungal Materials

Aseptic seedlings of *P. trichocarpa* were purchased from Nanjing Baisihe Biotechnology Co., Ltd. (Nanjing, China). They were grown on Woody Plant Medium (WPM) in glass culture bottles under a long-day photoperiod (16 h of light, 8 h of darkness) at 25 °C. The light intensity was 3000 lux [31,32].

*L. bicolor* S238N was provided by Professor Yahua Chen of Nanjing Agricultural College, which was grown on Potato Dextrose Agar (PDA) medium at 25 °C [33].

*B. dothidea* CXY001 was preserved at the Forest Disease Laboratory of the Forestry College of Northwest A&F University and activated on PDA medium at 28 °C [34].

### 2.2. L. bicolor–P. trichocarpa–B. dothidea Coculturing in Two Sandwich Culture Systems

The established method of *L. bicolor*–*P. trichocarpa* in vitro culture system [32] was used with some modifications. Mycelium of *L. bicolor* was cultivated for 14 d on PDA medium, which contained 15 g·L^−1^ agar. Stem cuttings from in vitro *P. trichocarpa* (about 1 cm in length) were precultured on WPM medium containing 0.5 mg·L^−1^ indole-3-acetic acid for 14 d to synchronize rhizogenesis. One side of the 9 × 9 cm binary Petri dish had 7 mL low-sugar (3% glucose) WPM medium to cultivate the root and *L. bicolor* mycelium, while the stem and leaves were on the other side without culture medium. Cellophane containing *L. bicolor* mycelium was put on the medium before transferring the plant tissue, while cellophane with the blank medium served as control. Cultures were arranged vertically, and the lower part of the dish was covered with a small black plastic bag. Those poplars were cultured under a 16 h·d^−1^ light photoperiod at 25 °C for 3 weeks.

After 3 weeks of symbiosis between *L. bicolor* and *P. trichocarpa*, the stems were infected with *B. dothidea* in a sterile environment. The method of inoculation with *B. dothidea* was to make a wound on the stem and then inoculate *B. dothidea* cake under aseptic conditions, similar to the method of Li et al. [34]. Agar disks containing *B. dothidea* fungus (6 mm) and sterile PDA medium disks (6 and 10 mm, 10 mm medium disks were placed under the stem for support) were prepared for the follow-up experiment. The epidermis of the central section of the stem was scratched, and the wound was exposed to an agar disk containing *B. dothidea* mycelium. After being sealed again, those poplars were cultured for 72 h under the conditions mentioned above.

The treatments were (1) non-fungus control (NN); (2) inoculation with *L. bicolor* and *B. dothidea* (EB); (3) inoculation with *B. dothidea* but no *L. bicolor* (NB); and (4) inoculation with *L. bicolor* but no *B. dothidea* (EN). Each treatment was replicated three times (two plants’ roots were combined into one replicate). The samples’ roots were quick-frozen with liquid nitrogen and ground into powder in a pre-cooled mortar, then put into a pre-cooled cryotube and stored at −80 °C in a refrigerator for subsequent testing.

### 2.3. Estimation of Peroxidase (POD) and L-phenylalanine Ammonia-Lyase (PAL)

POD activity was determined as described by Fang and Kao [35] and calculated from the rise in absorbance at 470 nm. The activities of POD were expressed as μg·g^−1^·FW·min^−1^.

PAL activity was measured according to Sreelakshmi and Sharma [36]. The absorbance was measured at 290 nm. The activities of PAL were expressed as U·g^−1^·FW·h^−1^.

### 2.4. Content of Malondialdehyde (MDA)

MDA was assayed according to the method described by Kramer et al. [37]. The content of MDA was expressed as μmol·g^−1^·FW.

### 2.5. RNA Extraction, Transcriptome Sequencing, and Bioinformatics Analysis

Total RNA was extracted using the E.Z.N.A Plant RNA Kit R6827-01 (Omega Bio-Tek, Norcross, GA, USA). The RNA samples were accepted when the 260/280 ratio was 1.9–2.1 using a Nano Photometer^®^ spectrophotometer (IMPLEN, CA, USA) and the RIN value (RNA integrity number) was >6.0 using an RNA Nano 6000 Assay Kit of the Bioanalyzer 2100 system (Agilent Technologies, Santa Clara, CA, USA). The clean reads after quality control were compared to the reference genome (https://ftp.ncbi.nlm.nih.gov/genomes/all/GCF/000/002/775/GCF_000002775.4_Pop_tri_v3/GCF_000002775.4_Pop_tri_v3_genomic.fna.gz, accessed: 10 December 2020) using Hisat2 (version 2.2.1) software [38]. The featureCounts tool in Subread (version 2.0.1) software [39] was used to count the number of reads covered from start to finish for each gene based on the location information of the gene alignment on the reference genome. Expression levels were estimated by transcripts per kilobase of exon model per million mapped reads (TPM). Differential expression analysis of control/treatment (two biological replicates per condition) was performed using the DESeq2 R package (version 1.32.0) [40] according to |log2 (Fold Change)| > 1 & padj < 0.05 for screening differentially expressed genes (DEGs). Significantly, DEGs were used for weighted correlation network analysis (WGCNA) using the WGCNA R package (version 1.69) [41], the soft thresholding powers value was 13, and the rest of the parameters were set according to the default parameters. Gene Ontology (GO) [42] and Kyoto Encyclopedia of Genes and Genomes (KEGG) [43] enrichment analysis of DEGs was implemented according to the default parameters by the clusterProfiler R package (version 4.0.0) [44]. The comparison of each treatment is expressed as control/treatment. The RNA-seq datasets using the Illumina-Solexa platform are available from the NCBI Sequence Read Archive database (SRA; http://www.ncbi.nlm.nih.gov/sra, accessed: 10 December 2020) under project number accession PRJNA683943.

### 2.6. The Quantitative Real-Time PCR (qRT-PCR)

Five genes (disease resistance protein RPM1, calmodulin-like protein 1, pentatricopeptide repeat-containing protein At3g18110, pathogenesis-related genes transcriptional activator PTI6, respiratory burst oxidase homolog protein B), mainly from plant–pathogen interaction-related pathways, were randomly selected and tested using quantitative real-time PCR (qRT-PCR) as described by Zhang et al. [45]. Two housekeeping genes (peptidyl-prolyl cis-trans isomerase 1, elongation factor 1-alpha-like) served as the reference genes [46,47]. All gene-specific primers in this study were designed using the NCBI Primer-BLAST (Table S1). The qRT-PCR reaction was conducted by the CF96X Real-time PCR system (Bio-Rad, Hercules, CA, USA). Each reaction mixture was 10 µL, containing 1 µL diluted cDNA template, 0.5 µL forward and reverse primers (10 mmol·L^−1^), 5 µL ChamQ SYBR qPCR Master Mix (Vazyme, Nanjing, China), and 3 µL sterilized ddH_2_O. The three-step qRT-PCR was run as follows: 3 min denaturation at 95 °C, 40 cycles of denaturation at 95 °C for 10 s, annealing at the annealing temperature (annealing temperature in Table S1) for 10 s, extension at 72 °C for 20 s, followed by heating from 65 to 95 °C at a rate of 0.5 °C every 5 s. All samples were amplified in duplicate from the same RNA preparation, and the mean value was considered. The relative expression of each target gene was calculated according to the 2^−^^△△Ct^ protocol [48].

### 2.7. Statistical Analysis

All experiments were repeated at least three times. All results are expressed as the mean ± standard error (SE) in tables and figures. Two-way analysis of variance (ANOVA) and Tukey’s tests using SPSS software (Version 26.0, SPSS Inc., Chicago, IL, USA) evaluated significant differences across all parameters.

## 3. Results

### 3.1. Enzyme Activity Analysis

As shown in Table 1, inoculation with *L. bicolor* significantly (*p* < 0.01) increased the activities of POD, PAL, and MDA content, suggesting that the infection with *L. bicolor* changed the reactive oxygen species content of the roots. Inoculation with *B. dothidea* also significantly (*p* < 0.05) increased the activities of POD and PAL and MDA content, indicating that disease stress affected gene expression. Inoculation with *L. bicolor* and *B. dothidea* extremely significantly (*p* < 0.01) reduced the MDA content of the roots, which indicated that *L. bicolor* could significantly reduce the effects of reactive oxygen species on the roots under disease stress.

### 3.2. Analysis of Differentially Expressed Genes between Different Treatments

As shown in Table 2, the number of down-regulated DEGs was more than up-regulated DEGs in NN/EN. The infection with *B. dothidea* caused the disease resistance of the roots, and the number of up-regulated DEGs was more than that of the down-regulated DEGs. EN/EB had the greatest number of DEGs among the four comparison groups, indicating that maybe the disease resistance in the mycorrhizal *P. trichocarpa* was stronger. Compared with NN/EN, the number of up-regulated and down-regulated DEGs in EN/EB increased by 148% and 52%, respectively. In NB/EB, the number of up-regulated DEGs decreased by 66% as compared to down-regulated ones, which might be due to the root needing to maintain symbiosis during disease.

**Figure 1:**
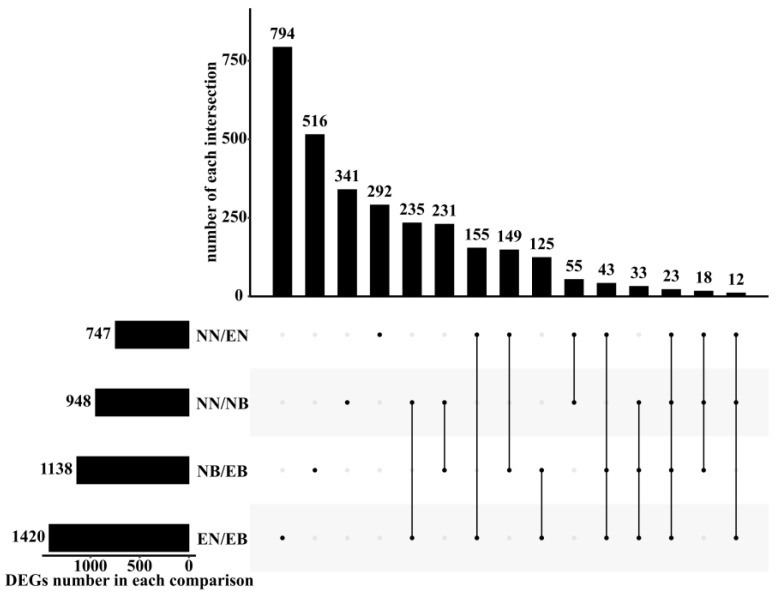
Upset diagram of differentially expressed genes. DEGs number in each comparison group represents the number of all DEGs in each comparison group; the number of each intersection represents the total number of DEGs in each comparison group; a point on the abscissa represents the number of unique DEGs in each comparison group; the line of multiple dots on the abscissa indicates the number of DEGs identified by the multiple comparison groups of the line.

A total of 3022 DEGs were identified in the four groups (Figure 1). In the comparison group with and without *L. bicolor* (NN/NB, EN/EB), *B. dothidea* regulated a total of 303 DEGs. These might be the main genes in the roots of *P. trichocarpa* in response to *B. dothidea* infection. However, these genes did not exceed half of the total DEGs of NN/NB or EN/EB, showing the variable mechanism of mycorrhizal *P. trichocarpa* roots in response to *B. dothidea* infection.

**Figure 2:**
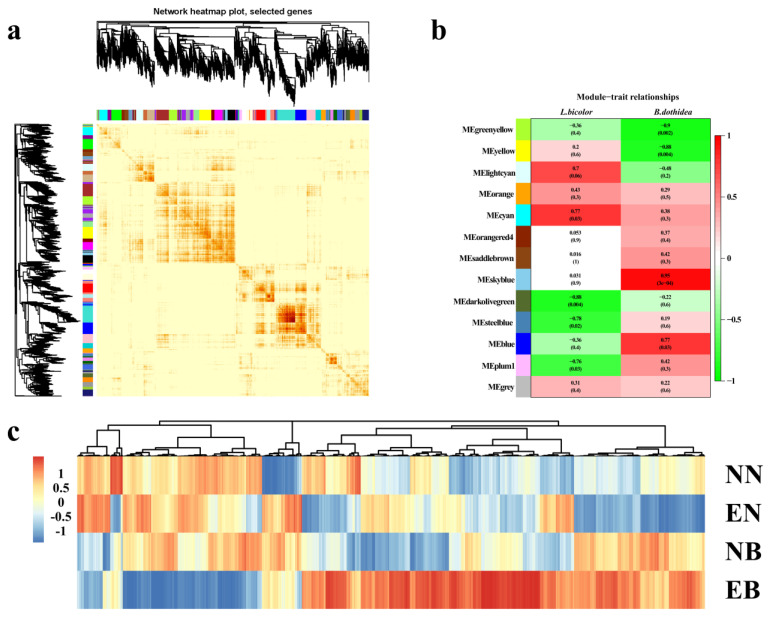
(**a**) The correlation heat map of the genes between the modules. (**b**) The heat map of the correlation between each module and the trait. Pearson’s correlation coefficients between modules and fungal inoculation are shown, accompanied by the corresponding *p* value in brackets. From red to green, the correlation probability is from high to low. Each module is identified by color. (**c**) A heat map of the expression levels of 661 DEGs in the four groups. From red to blue, the expression level was from high to low.

As shown in Figure 2, 3022 DEGs were divided into different modules according to different expression patterns. In 13 modules, the expression patterns of 661 DEGs in the “MEorange”, “MEcyan”, “MEgrey”, “MEorangered4”, “MEsaddlebrown”, and “MEskyblue” modules were positively correlated with the inoculation of *L. bicolor* and *B. dothidea* (Figure 2b). It indicated that these genes might be regulated by *L. bicolor* and participate in the process of *P. trichocarpa* in response to infection with *B. dothidea*.

### 3.3. DEGs Enrichment Analysis

**Figure 3:**
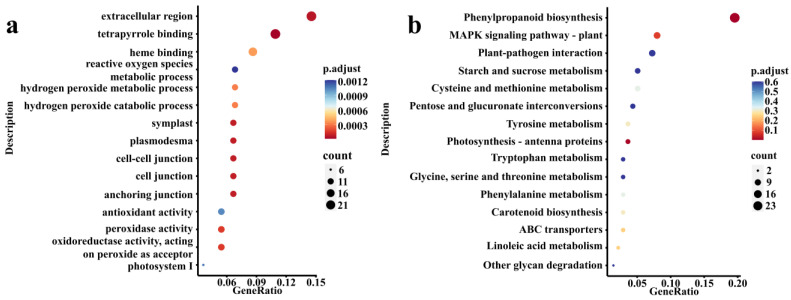
(**a**) The scatter map of GO enrichment analysis on 661 DEGs obtained by WGCNA. Sorted by p.adjust (from 0 to 1), the top 15 GO enrichment results were selected to display. (**b**) The scatter map of KEGG enrichment analysis on 661 DEGs obtained by WGCNA. Sorted by p.adjust (from 0 to 1), the top 15 KEGG enrichment results were selected to display. Gene ratio: A score, the numerator is the number of genes enriched in this GO entry and the denominator is the number of input genes for enrichment analysis, which can be the genes obtained by differential expression analysis; count: enter the number of genes enriched to this GO entry in the genes for enrichment analysis; p.adjust: corrected *p* value. For the complete GO and KEGG enrichment results, please view Tables S3 and S4.

GO enrichment analysis showed that 661 DEGs were all enriched (p.adjust = 1) in 770 GO terms, and significantly enriched (p.adjust < 0.05) in 51 GO terms (Figure 3a). Most of these GO terms were related to reactive oxygen metabolism (“peroxidase activity”, “hydrogen peroxide metabolic process”, and “reactive oxygen species metabolic process”), hormones (“methyl salicylate esterase activity”, “abscisic acid binding”, and “methyl jasmonate esterase activity”), and other related GO terms. KEGG enrichment analysis showed that all 661 DEGs were enriched (p.adjust = 1) in 71 KEGG pathways, which were significantly enriched (p.adjust < 0.05) in the two metabolic pathways “Phenylpropanoid biosynthesis” and “Photosynthesis-antenna proteins” (Figure 3b). *L. bicolor* might participate in the process of *P. trichocarpa* in response to infection with *B. dothidea* by affecting the expression of genes in these pathways.

### 3.4. Analysis of Gene Expression Patterns Related to Signal Transduction Induced by L. bicolor

**Figure 4:**
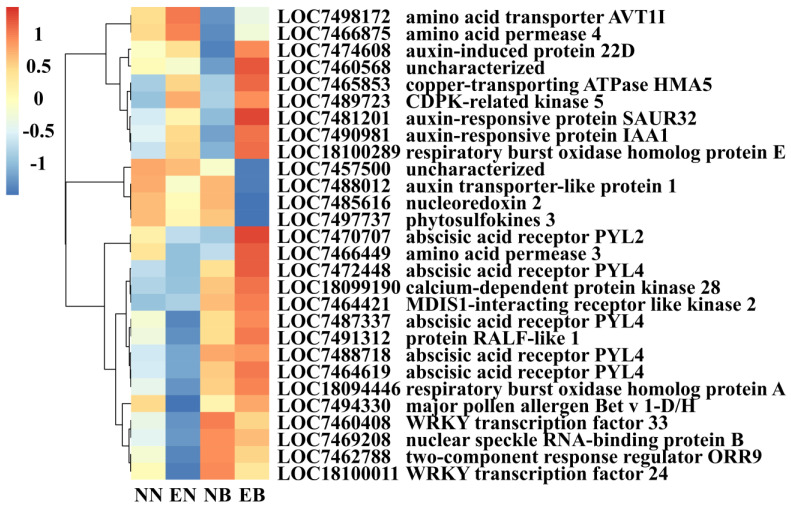
Heat map of DEGs related to signal transduction pathway.

Figure 3b shows that the changes in gene expression in the roots of *P. trichocarpa* in response to disease stress under the conditions of inoculation and non-inoculation with *L. bicolor* were different, and this change was often regulated by signal molecules. In GO enrichment analysis, signal transduction-related DEGs were mainly enriched in “abscisic acid-activated signaling pathway”, “hormone-mediated signaling pathway”, “signaling receptor activity”, “auxin-activated signaling pathway”, “signaling receptor activator activity”, “calcium-mediated signaling”, “second-messenger-mediated signaling”, “signaling receptor binding”, and “intracellular signal transduction”. In KEGG enrichment analysis, signal transduction-related DEGs were enriched in “MAPK signaling pathway-plant” and “plant hormone signal transduction”. A total of 28 DEGs were enriched in these pathways (Figure 4). Among these DEGs, four were related to auxin. The expression of LOC7474608 (auxin-induced protein 22D), LOC7481201 (auxin-responsive protein SAUR32), and LOC7490981 (auxin-responsive protein IAA1) was down-regulated in NB but was up-regulated in EN, and the expression level was further increased in EB due to the influence of *L. bicolor*. LOC7470707 (abscisic acid receptor PYL2), LOC7472448 (abscisic acid receptor PYL4), LOC7487337 (abscisic acid receptor PYL4), LOC7488718 (abscisic acid receptor PYL4), and LOC7464619 (abscisic acid receptor PYL4) were related to abscisic acid. In contrast to auxin, the induced expression of *L. bicolor* was inhibited in EN, while LOC7472448, LOC7487337, LOC7488718, and LOC7464619 were increased in NB, while *B. dothidea* inhibited the expression of LOC7470707. In EB, *L. bicolor* could induce a further increase in the expression of these DEGs.

LOC18100289 (respiratory burst oxidase homolog protein E) and LOC18094446 (respiratory burst oxidase homolog protein A) belong to the family of respiratory burst oxidase homolog (Rboh) proteins. The single inoculation with *L. bicolor* inhibited the expression of LOC18094446 and increased the expression of LOC18100289. The opposite was true when inoculating with *B. dothidea* alone. In EB, *L. bicolor* would further increase the expression level of LOC18094446, and the expression level of LOC18094446 was also increased by the influence of *B. dothidea*, but the influence of *L. bicolor* was lower than the expression level when inoculated with *B. dothidea* alone. LOC7460408 (WRKY transcription factor 33) and LOC18100011 (WRKY transcription factor 24) belonged to the family of WRKY transcription factors. They were inhibited by *L. bicolor* in EN. The infestation of *B. dothidea* induces an increase in their expression, but their expression was lower than that in NB. Perhaps *L. bicolor* could help *P. trichocarpa* resist the infection with *B. dothidea* by regulating the expression of these genes.

### 3.5. Analysis of the Expression Pattern of Disease Resistance-Related and Antioxidant-Related DEGs Induced by L. bicolor

**Figure 5:**
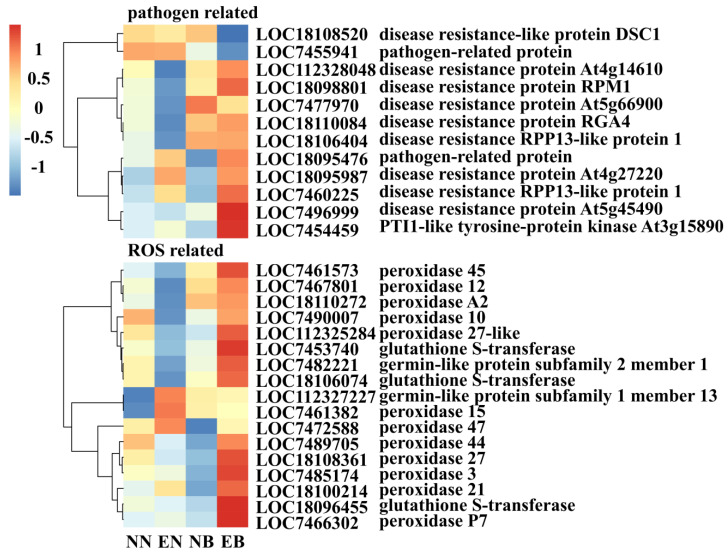
Heat map of DEGs related to disease resistance and antioxidant.

Out of all 661 DEGs, a total of 12 disease resistance-related DEGs were found (Figure 5). Inoculation with *L. bicolor* inhibited the expression of LOC112328048 (disease resistance protein At4g14610), LOC18098801 (disease resistance protein RPM1), LOC7477970 (disease resistance protein At5g66900), LOC18110084 (putative disease resistance protein RGA4), and LOC18106404 (putative disease resistance RPP13-like protein 1), while single inoculation with *B. dothidea* increased the expression of these genes. In EB, the inoculation with *L. bicolor* could promote the expression of the remaining DEGs, except for LOC7477970. The expression of LOC18095476 (pathogen-related protein), LOC18095987 (probable disease resistance protein At4g27220), LOC7460225 (putative disease resistance RPP13-like protein 1), LOC7496999 (disease resistance protein At5g45490), and LOC7454459 (PTI1-like tyrosine-protein kinase At3g15890) were inhibited in NB, but under the influence of *L. bicolor*, the expression level increased. These results imply that *L. bicolor* changes the expression of disease-resistant genes, thereby protecting *P. trichocarpa* against infection with *B. dothidea*.

A total of 17 antioxidant enzyme-related DEGs were found, belonging to the POD family, germin-like protein (GLP) subfamily, and glutathione S-transferase (GST) family. Compared with NB, the expression level of 15 DEGs in EB was increased by the influence of *L. bicolor*. LOC112327227 (germin-like protein subfamily 1 member 13), LOC7461382 (peroxidase 15), and LOC7472588 (peroxidase 47) had the highest expression levels in EN, but inoculation with *L. bicolor* reduced the expression levels of these three DEGs.

### 3.6. The qRT-PCR Verification

LOC18098801 (disease resistance protein RPM1), LOC7483121 (calmodulin-like protein 1), LOC18106973 (pentatricopeptide repeat-containing protein At3g18110), LOC7494656 (pathogenesis-related genes transcriptional activator PTI6), and LOC18098678 (respiratory burst oxidase homolog protein B) were selected and analyzed by qRT-PCR to validate the RNA-Seq data. Their trends were similar to those of the transcriptome (Figure S1).

## 4. Discussion

In the process of resisting the infection of pathogenic fungi, plants have evolved a set of sophisticated and efficient defense mechanisms [49]. After the immunoreceptors on the surface of plant cells recognize the pathogenic fungus, they produce disease-resistant signals, which are transmitted to the whole body, changing the level of gene expression and producing anti-disease substances to inhibit or kill pathogenic fungi [50,51]. Inoculation with mycorrhizal fungi and pathogenic fungi will cause a defensive response in the non-infected parts [52]. The expression levels of genes change after the roots are infected by mycorrhizal fungi, which indirectly changes the gene expression level of stems and leaves [22,53]. This may be one of the important ways for mycorrhizal fungi to help the stems and leaves of the host resist the invasion of pathogens. The roots also foster disease resistance after pathogens invade the stems and leaves [54].

Similar to Zhan et al. [20], in our study, whether inoculated with *L. bicolor* or *B. dothidea*, the activity of disease-resistant enzymes (POD and PAL) in roots increased. After inoculation with the two fungi, the activities of POD and PAL in *P. trichocarpa* were significantly increased. Changes in enzymes activity are caused by a series of changes in gene expression. This proved to a certain extent the hypothesis put forward by this research; that is, *L. bicolor* changes the gene expression pattern of the roots of *P. trichocarpa* under disease stress and participates in the disease resistance of *P. trichocarpa*. With the help of WCGNA, a total of 661 DEGs were found to be affected by *L. bicolor* and *B. dothidea*. The different expression patterns of these DEGs under different treatments further confirmed the hypothesis of this study. Through the in-depth analysis of these DEGs, it was revealed that *L. bicolor* participates in the resistance of *P. trichocarpa* against *B. dothidea* infection by regulating the expression of root genes.

The transmission of disease resistance signals is one of the key steps in plant disease resistance response [55]. In this process, signal molecules bind to downstream receptors to regulate the expression of defense response genes. Disease resistance signal transduction pathways in plants include the hormone signal transduction pathway, the Ca^2+^ signal transduction pathway, and the ROS signal transduction pathway [56].

Auxin plays an important role in regulating the development of the plant root system and vascular system and establishing a symbiotic relationship between mycorrhizal fungi and roots [32]. ECMF changes the level of host plant auxin, thereby inducing lateral roots, restricting the growth of main roots, and making the roots grow horizontally while inhibiting host root hairs [57]. These strategies to change the morphological structure of the root system increased the infection point of mycelium and promoted the establishment of the ECMF symbiotic relationship [58,59]. It also directly or indirectly participates in the defense of plants against pathogens [60,61]. Auxin is generally considered to play a negative regulatory role in the process of plant disease resistance [62,63]. IAA treatment of rice will reduce the resistance of rice to *Xanthomonas oryzae pv. oryzae* [64]. In our study, infection with *B. dothidea* inhibited the expression of auxin-induced protein 22D (LOC7474608), auxin-responsive protein SAUR32 (LOC7481201), and auxin-responsive protein IAA1 (LOC7490981) in the roots. According to the idea that the increase in auxin content inhibits plant disease resistance, in the case of inoculation with *B. dothidea*, the roots of *P. trichocarpa* may inhibit the expression of these three genes to improve disease resistance. The results of the inoculation with *L. bicolor* were the opposite. Regardless of whether the disease occurred, the expression levels of these three genes were induced to increase. Mycorrhiza secretes trace hormones to regulate the growth and development of plants [65], which may be the reason for the increased expression of those three genes. At the same time, after the onset of the disease, the expression of genes further increased. Studies have found that biocontrol fungi and pathogenic fungi can increase the expression of some genes in the auxin pathway when they infect plants at the same time [63,66]. Therefore, the increase in the expression of these three genes may be one of the ways that *L. bicolor* participates in the resistance to *B. dothidea* infection with *P. trichocarpa*. The PYL (Pyrabactin-like) family is an ABA receptor that senses ABA changes in plants and plays an important role in the response to biotic and abiotic stresses [67]. Chen [68] found that overexpression of *SiPYL4* in *Arabidopsis thaliana* can increase disease resistance to *Macrophomina phaseolina* and prolong survival time. Our results were similar to those. In NB, the expression of four DEGs out of five abscisic acid signal transduction DEGs were up-regulated. These four DEGs belonged to *PYL4*. However, in EB, the inoculation with *L. bicolor* further increased the expression of these four DEGs. This may be a way to improve the disease resistance of *P. trichocarpa*. In addition, inoculation with *L. bicolor* also increased the expression of *PYL2*, but the effect of *PYL2* on disease stress is still unclear, and further research is needed.

The change of ROS content in plants is also an important aspect of inducing plant disease resistance [69,70,71,72,73]. When plants feel the stimulation of hormones and pathogens, the cells produce Ca^2+^ and combine with Rboh to activate NADPH (nicotinamide adenine dinucleotide phosphate) oxidase, catalyzing the production of a large amount of ROS to inhibit the growth of pathogens [68]. Yoshioka et al. [74] found that *NbRbohA* and *NbRbohB* were involved in the production of H_2_O_2_ and resistance to pathogenic oomycete (*Phytophthora infestans*) in tobacco disease resistance. Qin et al. [75] studied the changes in the expression of the Rboh family after citrus infection by *B. dothidea* and found that the expression of *Rboh E* in disease-resistant varieties was lower than that of susceptible varieties, indicating that it is involved in plant disease resistance. In our study, inoculation with *B. dothidea* up-regulated *Rboh A* and *Rboh E*, but the expression of these two DEGs in EB was higher than that in NB. It showed that *L. bicolor* increased the expression of these two genes and participated in the disease resistance of *P. trichocarpa*. At the same time, studies have shown that there are wound response elements in the promoter region of *Rboh E*. W-box interacts with members of the WRKY transcription factor family and plays a key role in biological stress [76,77]. Many studies have shown that most of the WRKY family genes are involved in plant disease resistance [78]. Although inoculation with *L. bicolor* increased the expression of *Rboh E*, the expression of *WRKY33* and *WRKY24* in EB were slightly lower than those in NB. It might be that *L. bicolor* slightly suppressed their expression to maintain a symbiotic relationship.

After a series of transductions, the disease-resistant signal finally acted on the direct disease-resistant protein and antioxidant enzyme synthesis gene to deal with the invasion of pathogenic fungi [49]. Inoculation with *L. bicolor* changed the expression of signal transduction pathway genes and finally acted on disease-resistant proteins and antioxidant enzyme genes, changing their expression levels. Both *RPM1* and *RPP13* are important members of the disease resistance network in plants, and they play an important role in identifying pathogens and regulating downstream disease resistance [79,80,81]. In our study, inoculation with *L. bicolor* mainly affected 12 genes related to direct disease resistance, among which inoculation with *L. bicolor* further increased the expression of *RPM1* and *RPP13*. *L. bicolor* might participate in disease resistance through the regulation of direct disease resistance proteins.

The invasion of *B. dothidea* and *L. bicolor* will change the original structure of the plant, produce varying degrees of damage, and release ROS [20]. A small amount of ROS helps to activate the plant’s disease resistance response, but excessive ROS can damage cell membranes [82], so plants need to increase the activity of antioxidant enzymes to remove excess ROS. POD, GLP, and GST are important enzymes for removing ROS in plants [83]. Through transcriptome analysis, it was found that during the period of disease stress, *L. bicolor* induced an increase in the expression of POD, GST, and GLP genes. Perhaps the increase in the expression of these genes caused the increase in the activity of the corresponding protein, which reduced the ROS content in the plant, thereby alleviating the damage caused by the disease stress.

## 5. Conclusions

In this study, transcriptome analysis technology was used to explore the gene expression changes in the mycorrhizal *P*. *trichocarpa* roots under poplar canker stress. Our research showed that inoculation with *L. bicolor* changed the expression pattern of 661 genes in the roots. These genes were involved in many pathways such as signal transduction, reactive oxygen metabolism, and plant–pathogen interaction. The expression of these genes was changed due to inoculation with *L. bicolor*, which suggests that *L. bicolor* affects the disease resistance of *P*. *trichocarpa*. Our results not only provide a theoretical basis for revealing the molecular mechanism of mycorrhizal fungi improving the resistance of *P*. *trichocarpa* to poplar canker but also provide a theoretical basis for the development and application of biological agents.

## Figures and Tables

**Table 1:** Enzyme activity under different treatments.

Treatment	POD	PAL	MDA
NN	1054.02 ± 325.66 c	5678.41 ± 1428.19 c	14.30 ± 3.90 d
EN	3516.56 ± 484.89 ab	16600.94 ± 779.80 ab	35.49 ± 5.53 c
NB	2330.89 ± 465.16 bc	10399.50 ± 2842.16 bc	128.66 ± 11.48 a
EB	5252.68 ± 1370.74 a	23236.47 ± 5403.70 a	60.68 ± 8.61 b
*L. bicolor*	**	**	**
*B. dothidea*	*	*	**
*L. bicolor* & *B. dothidea*	ns	ns	**

NN: non-fungus control; EN: inoculation with *L. bicolor* but no *B. dothidea*; NB: inoculation with *B. dothidea* but no *L. bicolor*; EB: inoculation with *L. bicolor* and *B. dothidea*. FW: fresh weight. Data expressed as mean ± standard error (*n* = 3). Different lowercase letters indicate significant differences between the means by Tukey’s test (*p* < 0.05); “*” indicates that the interaction is significant (*p* < 0.05); “**” indicates that the interaction is extremely significant (*p* < 0.01); “ns” indicates no interaction (*p* ≥ 0.05). The activities of POD were expressed as μg·g^−1^·FW·min^−1^. The activities of PAL were expressed as U·g^−1^·FW·h^−1^. The content of MDA was expressed as μmol·g^−1^·FW.

**Table 2:** The number of differentially expressed genes (DEGs) in the four comparison groups.

Comparisons (Control/Treatment)	All DEGs	Up Regulated DEGs	Down Regulated DEGs
NN/EN	747	297	450
NN/NB	948	734	214
EN/EB	1420	736	684
NB/EB	1138	288	850

## Data Availability

The data that support the findings of this study are available on request from the corresponding author.

## Supplementary Materials

The following are available online at https://www.mdpi.com/article/10.3390/jof7121024/s1: Figure S1—The qRT-PCR verification of DEGs was identified by transcriptome analysis. Table S1—qRT-PCR primers used in this study. Table S2—Summary of transcriptome sequencing results. Table S3—Results of KEGG enrichment analysis on 661 DEGs obtained by WGCNA. Table S4—Results of GO enrichment analysis on 661 DEGs obtained by WGCNA.
