# Supplementary material for: Effects of Laccaria bicolor on Gene Expression of Populus trichocarpa Root under Poplar Canker Stress
Source: J Fungi (Basel). 2021 Nov 29;7(12):1024. doi: 10.3390/jof7121024 (PMC8703858; doi:10.3390/jof7121024)
Supplement: Supplementary file 1 [file jof-07-01024-s001.zip › jof-1416630-supplementary.pdf]

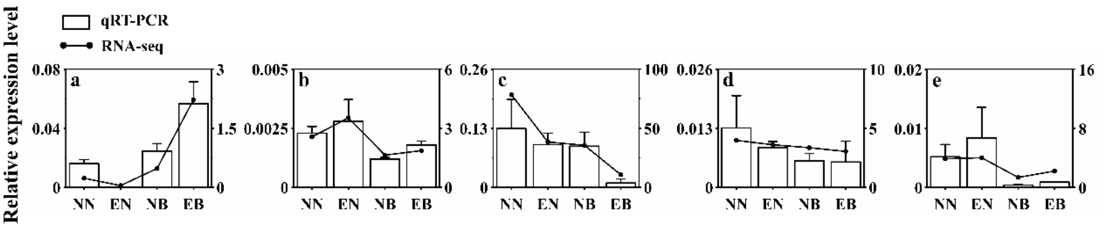

Figure S1. The qRT-PCR verification of DEGs was identified by transcriptome analysis. The left Y-axis belongs to the qRT-PCR, and the right Y-axis belongs to the RNA-seq. a: LOC18098801 (*disease resistance protein RPM1*); b: LOC18106973 (*pentatricopeptide repeat-containing protein At3g18110*); c: LOC7494656 (*pathogenesis-related genes transcriptional activator PTI6*); d: LOC18098678 (*respiratory burst oxidase homolog protein B*); e: LOC7483121 (*calmodulin-like protein 1*). Standard deviation is represented by the error bars (n=3).

Table S1: qRT-PCR primers used in this study.

Gene ID	Forward Primer (5'->3')	Reverse Primer (5'->3')	Tm (°C)	Gene symbol
LOC18098801	TGGTCACAGCAAGAATCCCA	TCCCAAGCAAAAACATCCTGTAA	56.5	disease resistance protein RPM1
LOC7483121	GGGAGAAGAGGCAAAGCTGTA	AAGGCCCTCAGTGCTGATTT	59.2	calmodulin-like protein 1
LOC18106973	GGCACTTGGACTAGACCTATTCAT	GCTCACATATTATTTTCCCACGGTT	56.5	pentatricopeptide repeat-containing protein At3g18110
LOC7494656	TTGGCTTACGGTGACGTTGA	AACATGCCACGTCGTGTTTG	56.5	pathogenesis-related genes transcriptional activator PTI6
LOC18098678	TGACCCGTGCTTTTCGATCA	GTTGCGATGTCCAGTCACCT	56.5	respiratory burst oxidase homolog protein B
LOC7477866 [1]	GGCTAATTTTGCCGATGAGA	ACGTCCATCCCTTCAACAAC	56.5	peptidyl-prolyl cis-trans isomerase 1
LOC112328984 [2]	ATTGACAGGCGGTCTGGTAAGGAA	AAACGACCAAGTGGAGGATACGCT	56.0	elongation factor 1-alpha-like

Note: [1] The accession number in the original article is BU875027; [2] The accession number in the original article is GQ253565.1.

Table S2: Summary of transcriptome sequencing results.

Sample	Raw Rreads	Clean Reads	Clean Bases (G)	Q20 (%)	Q30 (%)	GC (%)	Total Map Rate (%)
EB1	45253696	43272406	6.49	98.46	94.93	44.67	86.78
EB2	47254952	45116300	6.77	98.38	94.78	45.67	74.93
EB3	47775218	45615970	6.84	98.26	94.48	45.12	81.57
EN1	43446220	41452280	6.22	98.41	94.86	45.65	76.40
EN2	50546564	48119706	7.22	98.43	94.88	44.73	85.60
EN3	48269278	45782372	6.87	98.41	94.93	48.35	43.59
NB1	46107376	43892640	6.58	98.41	94.83	43.90	96.52
NB2	46389626	44273604	6.64	98.36	94.69	43.95	96.56
NB3	45964794	43624210	6.54	98.34	94.66	44.29	94.05
NN1	45895672	43954528	6.59	98.54	95.08	43.96	96.77
NN2	45456422	43557190	6.53	98.03	93.88	44.01	96.15
NN3	47501772	45570736	6.84	98.37	94.70	43.91	96.70

Sample: sample name; raw reads: the number of reads in the original data; clean reads: the number of reads of the original data after quality filtering; clean bases: the number of bases after filtering the original

data (clean base=clean reads*150 bp); Q20: the percentage of bases with a Phred value greater than 20 to the total bases (Phred=-10log10(e)); Q30: the percentage of bases with a Phred value greater than 30 to the total bases; GC: the percentage of G and C in the four bases in clean reads; total map rate: percentage of reads aligned to the genome. An average of 46,655,133 raw reads was obtained with a Q20 value of 98.37% and a Q30 value of 94.73%. After quality filtering, an average of 6.68 G data was obtained, with an average of 44,519,329 clean reads and a GC content of 44.85%. The average total map rate of each sample was 85.47%.

Table S3: Results of KEGG enrichment analysis on 661 DEGs obtained by WGCNA.

ID	Description	p.adjust	Count
pop00940	Phenylpropanoid biosynthesis	9.53E-11	27
pop00196	Photosynthesis - antenna proteins	0.00611	5
pop04016	MAPK signaling pathway - plant	0.080819	11
pop00591	Linoleic acid metabolism	0.247816	3
pop02010	ABC transporters	0.247816	4
pop00350	Tyrosine metabolism	0.300403	5
pop00906	Carotenoid biosynthesis	0.300403	4
pop00360	Phenylalanine metabolism	0.325669	4
pop00270	Cysteine and methionine metabolism	0.325669	7
pop04626	Plant-pathogen interaction	0.601483	10
pop00500	Starch and sucrose metabolism	0.604506	7
pop00260	Glycine, serine and threonine metabolism	0.604506	4
pop00380	Tryptophan metabolism	0.604506	4
pop00511	Other glycan degradation	0.605974	2
pop00040	Pentose and glucuronate interconversions	0.605974	6
pop04075	Plant hormone signal transduction	0.645215	11
pop00010	Glycolysis / Gluconeogenesis	0.656741	6
pop00480	Glutathione metabolism	0.656741	5
pop00960	Tropane, piperidine and pyridine alkaloid biosynthesis	0.656741	2
pop00071	Fatty acid degradation	0.656741	3
pop00052	Galactose metabolism	0.674171	3
pop00945	Stilbenoid, diarylheptanoid and gingerol biosynthesis	0.674171	2
pop00950	Isoquinoline alkaloid biosynthesis	0.769834	2
pop00750	Vitamin B6 metabolism	0.834435	1
pop00531	Glycosaminoglycan degradation	0.889531	1
pop00450	Selenocompound metabolism	0.889531	1
pop00590	Arachidonic acid metabolism	0.889531	1
pop00061	Fatty acid biosynthesis	0.889531	2
pop00250	Alanine, aspartate and glutamate metabolism	0.889531	2
pop00780	Biotin metabolism	0.889531	1
pop04141	Protein processing in endoplasmic reticulum	0.889531	7
pop00410	beta-Alanine metabolism	0.889531	2
pop00670	One carbon pool by folate	0.889531	1

pop00904	Diterpenoid biosynthesis	0.889531	1
pop01040	Biosynthesis of unsaturated fatty acids	0.889531	1
pop00740	Riboflavin metabolism	0.889531	1
pop00053	Ascorbate and aldarate metabolism	0.889531	2
pop00941	Flavonoid biosynthesis	0.889531	2
pop00900	Terpenoid backbone biosynthesis	0.918582	2
pop00592	alpha-Linolenic acid metabolism	0.918582	2
pop00565	Ether lipid metabolism	0.918582	1
pop00600	Sphingolipid metabolism	0.918582	1
pop00620	Pyruvate metabolism	0.918582	3
pop01212	Fatty acid metabolism	0.918582	2
pop00910	Nitrogen metabolism	0.918582	1
pop00195	Photosynthesis	0.918582	2
pop01200	Carbon metabolism	0.918582	7
pop01230	Biosynthesis of amino acids	0.918582	6
pop00710	Carbon fixation in photosynthetic organisms	0.918582	2
pop00220	Arginine biosynthesis	0.918582	1
pop04712	Circadian rhythm - plant	0.951846	1
pop04130	SNARE interactions in vesicular transport	0.951846	1
pop00520	Amino sugar and nucleotide sugar metabolism	0.951846	3
pop00400	Phenylalanine, tyrosine and tryptophan biosynthesis	0.951846	1
pop00860	Porphyrin and chlorophyll metabolism	0.951846	1
pop00280	Valine, leucine and isoleucine degradation	0.951846	1
pop00564	Glycerophospholipid metabolism	0.951846	2
pop03060	Protein export	0.951846	1
pop00030	Pentose phosphate pathway	0.951846	1
pop00051	Fructose and mannose metabolism	0.951846	1
pop00240	Pyrimidine metabolism	0.951846	1
pop04144	Endocytosis	0.951846	3
pop00561	Glycerolipid metabolism	0.962602	1
pop00562	Inositol phosphate metabolism	0.962602	1
pop00630	Glyoxylate and dicarboxylate metabolism	0.965969	1
pop04145	Phagosome	0.991144	1
pop03040	Spliceosome	0.991144	2
pop01240	Biosynthesis of cofactors	0.991144	3
pop00190	Oxidative phosphorylation	0.991144	1
pop04120	Ubiquitin mediated proteolysis	0.991144	1
pop03010	Ribosome	0.999964	1

ID: KEGG pathway number. corrected p value. Count: the number of genes enriched in each pathway.

Table S4: Results of GO enrichment analysis on 661 DEGs obtained by WGCNA.

ID	Description	p.adjust	Count
GO:0042744	hydrogen peroxide catabolic process	0.000303	11

GO:0042743	hydrogen peroxide metabolic process	0.000303	11
GO:0072593	reactive oxygen species metabolic process	0.001203	11
GO:0032259	methylation	0.001315	12
GO:0006979	response to oxidative stress	0.001635	12
GO:0009768	photosynthesis, light harvesting in photosystem I	0.004288	5
GO:0009765	photosynthesis, light harvesting	0.007867	5
GO:0015849	organic acid transport	0.009111	8
GO:0080163	regulation of protein serine/threonine phosphatase activity	0.024198	5
GO:0016052	carbohydrate catabolic process	0.031066	11
GO:0010921	regulation of phosphatase activity	0.031066	5
GO:0018298	protein-chromophore linkage	0.031066	5
GO:0043666	regulation of phosphoprotein phosphatase activity	0.031066	5
GO:0046942	carboxylic acid transport	0.032921	4
GO:0009628	response to abiotic stimulus	0.050516	12
GO:0042221	response to chemical	0.050516	22
GO:0009738	abscisic acid-activated signaling pathway	0.050516	5
GO:0071215	cellular response to abscisic acid stimulus	0.050516	5
GO:0097306	cellular response to alcohol	0.050516	5
GO:0035304	regulation of protein dephosphorylation	0.050516	5
GO:0009314	response to radiation	0.050516	8
GO:0009416	response to light stimulus	0.050516	8
GO:0035303	regulation of dephosphorylation	0.05321	5
GO:0042537	benzene-containing compound metabolic process	0.061308	4
GO:0030245	cellulose catabolic process	0.064751	3
GO:0051275	beta-glucan catabolic process	0.064751	3
GO:0000272	polysaccharide catabolic process	0.065864	7
GO:0019684	photosynthesis, light reaction	0.065864	6
GO:0006952	defense response	0.065864	11
GO:0010033	response to organic substance	0.067546	18
GO:0015711	organic anion transport	0.086481	4
GO:0071310	cellular response to organic substance	0.086481	14
GO:0009725	response to hormone	0.095295	15
GO:0030243	cellulose metabolic process	0.095295	4
GO:0051273	beta-glucan metabolic process	0.095295	4
GO:0009719	response to endogenous stimulus	0.095295	15
GO:0009694	jasmonic acid metabolic process	0.095295	3
GO:0009755	hormone-mediated signaling pathway	0.09691	12
GO:0032870	cellular response to hormone stimulus	0.09691	12
GO:0071495	cellular response to endogenous stimulus	0.098015	12
GO:0009696	salicylic acid metabolic process	0.098015	3
GO:0044247	cellular polysaccharide catabolic process	0.098015	3
GO:0070887	cellular response to chemical stimulus	0.10263	15
GO:0044275	cellular carbohydrate catabolic process	0.105189	4
GO:0009737	response to abscisic acid	0.114288	5

GO:0097305	response to alcohol	0.114288	5
GO:0018958	phenol-containing compound metabolic process	0.116217	3
GO:0015979	photosynthesis	0.123184	8
GO:0009251	glucan catabolic process	0.125805	3
GO:0009733	response to auxin	0.125805	9
GO:0051336	regulation of hydrolase activity	0.161785	5
GO:0042545	cell wall modification	0.174141	4
GO:0071214	cellular response to abiotic stimulus	0.176499	2
GO:0104004	cellular response to environmental stimulus	0.176499	2
GO:0005975	carbohydrate metabolic process	0.176499	19
GO:0016042	lipid catabolic process	0.206661	5
GO:0006470	protein dephosphorylation	0.22537	5
GO:0019220	regulation of phosphate metabolic process	0.229062	5
GO:0051174	regulation of phosphorus metabolic process	0.229062	5
GO:0006865	amino acid transport	0.254997	4
GO:0006555	methionine metabolic process	0.254997	2
GO:0009086	methionine biosynthetic process	0.254997	2
GO:0010038	response to metal ion	0.254997	2
GO:0009734	auxin-activated signaling pathway	0.274506	6
GO:0071365	cellular response to auxin stimulus	0.274506	6
GO:0065009	regulation of molecular function	0.304111	7
GO:1901615	organic hydroxy compound metabolic process	0.304111	7
GO:1903825	organic acid transmembrane transport	0.304111	2
GO:1905039	carboxylic acid transmembrane transport	0.304111	2
GO:0031399	regulation of protein modification process	0.30589	5
GO:0016114	terpenoid biosynthetic process	0.321217	3
GO:1901701	cellular response to oxygen-containing compound	0.331241	6
GO:0009607	response to biotic stimulus	0.345238	5
GO:0071396	cellular response to lipid	0.382216	5
GO:0009408	response to heat	0.393811	3
GO:0006721	terpenoid metabolic process	0.412446	3
GO:0005976	polysaccharide metabolic process	0.412639	8
GO:0034605	cellular response to heat	0.412639	2
GO:0016311	dephosphorylation	0.428776	5
GO:0006012	galactose metabolic process	0.440412	2
GO:0005996	monosaccharide metabolic process	0.44227	5
GO:0030154	cell differentiation	0.44227	5
GO:0009699	phenylpropanoid biosynthetic process	0.46225	2
GO:0006720	isoprenoid metabolic process	0.463588	4
GO:0048869	cellular developmental process	0.470835	5
GO:0009132	nucleoside diphosphate metabolic process	0.477949	3
GO:0042026	protein refolding	0.477949	2
GO:0046148	pigment biosynthetic process	0.515122	5
GO:0043207	response to external biotic stimulus	0.516303	4

GO:0051707	response to other organism	0.516303	4
GO:0098542	defense response to other organism	0.516303	4
GO:0050790	regulation of catalytic activity	0.523411	6
GO:0044419	biological process involved in interspecies interaction between organisms	0.523411	4
GO:0009266	response to temperature stimulus	0.529001	3
GO:0000097	sulfur amino acid biosynthetic process	0.543782	2
GO:1901700	response to oxygen-containing compound	0.554251	7
GO:0010035	response to inorganic substance	0.554251	3
GO:0006091	generation of precursor metabolites and energy	0.554251	8
GO:0000096	sulfur amino acid metabolic process	0.554251	2
GO:0042440	pigment metabolic process	0.554251	5
GO:0033993	response to lipid	0.575386	5
GO:0009067	aspartate family amino acid biosynthetic process	0.575386	2
GO:0019318	hexose metabolic process	0.59007	4
GO:0008299	isoprenoid biosynthetic process	0.59007	3
GO:0045490	pectin catabolic process	0.593929	3
GO:0008652	cellular amino acid biosynthetic process	0.593929	5
GO:0006986	response to unfolded protein	0.593929	2
GO:0009636	response to toxic substance	0.593929	2
GO:0034620	cellular response to unfolded protein	0.593929	2
GO:0044550	secondary metabolite biosynthetic process	0.593929	2
GO:0048580	regulation of post-embryonic development	0.593929	2
GO:0006073	cellular glucan metabolic process	0.633976	4
GO:2000026	regulation of multicellular organismal development	0.648991	2
GO:0009066	aspartate family amino acid metabolic process	0.662107	2
GO:0044042	glucan metabolic process	0.662107	4
GO:0098656	anion transmembrane transport	0.662107	4
GO:0032787	monocarboxylic acid metabolic process	0.662107	7
GO:1901607	alpha-amino acid biosynthetic process	0.662245	4
GO:0071555	cell wall organization	0.662245	5
GO:0019751	polyol metabolic process	0.662245	2
GO:0046777	protein autophosphorylation	0.662245	3
GO:0050793	regulation of developmental process	0.662245	3
GO:1901605	alpha-amino acid metabolic process	0.662245	5
GO:0044262	cellular carbohydrate metabolic process	0.662245	6
GO:0006071	glycerol metabolic process	0.662245	1
GO:0006558	L-phenylalanine metabolic process	0.662245	1
GO:0009637	response to blue light	0.662245	1
GO:0015740	C4-dicarboxylate transport	0.662245	1
GO:0015743	malate transport	0.662245	1
GO:0019320	hexose catabolic process	0.662245	1
GO:0019400	alditol metabolic process	0.662245	1
GO:0031204	posttranslational protein targeting to membrane, translocation	0.662245	1
GO:0098661	inorganic anion transmembrane transport	0.662245	1

GO:1902221	erythrose 4-phosphate/phosphoenolpyruvate family amino acid metabolic process	0.662245	1
GO:0009718	anthocyanin-containing compound biosynthetic process	0.662245	3
GO:0010393	galacturonan metabolic process	0.662245	3
GO:0045488	pectin metabolic process	0.662245	3
GO:0051084	'de novo' posttranslational protein folding	0.662245	2
GO:0051085	chaperone cofactor-dependent protein refolding	0.662245	2
GO:0046283	anthocyanin-containing compound metabolic process	0.662245	3
GO:0016119	carotene metabolic process	0.662245	1
GO:0030388	fructose 1,6-bisphosphate metabolic process	0.662245	1
GO:0051259	protein complex oligomerization	0.662245	1
GO:0006006	glucose metabolic process	0.662245	2
GO:0006458	'de novo' protein folding	0.662245	2
GO:0009072	aromatic amino acid family metabolic process	0.662245	2
GO:0045229	external encapsulating structure organization	0.662245	5
GO:0046394	carboxylic acid biosynthetic process	0.662245	7
GO:0006096	glycolytic process	0.662245	2
GO:0006165	nucleoside diphosphate phosphorylation	0.662245	2
GO:0006757	ATP generation from ADP	0.662245	2
GO:0009135	purine nucleoside diphosphate metabolic process	0.662245	2
GO:0009179	purine ribonucleoside diphosphate metabolic process	0.662245	2
GO:0046031	ADP metabolic process	0.662245	2
GO:0046939	nucleotide phosphorylation	0.662245	2
GO:0000281	mitotic cytokinesis	0.662245	1
GO:0006620	posttranslational protein targeting to endoplasmic reticulum membrane	0.662245	1
GO:0006835	dicarboxylic acid transport	0.662245	1
GO:0008300	isoprenoid catabolic process	0.662245	1
GO:0009423	chorismate biosynthetic process	0.662245	1
GO:0009809	lignin biosynthetic process	0.662245	1
GO:0009833	plant-type primary cell wall biogenesis	0.662245	1
GO:0019722	calcium-mediated signaling	0.662245	1
GO:0046185	aldehyde catabolic process	0.662245	1
GO:0061640	cytoskeleton-dependent cytokinesis	0.662245	1
GO:0009813	flavonoid biosynthetic process	0.662245	3
GO:0055085	transmembrane transport	0.662245	7
GO:0009812	flavonoid metabolic process	0.662245	3
GO:0009185	ribonucleoside diphosphate metabolic process	0.662245	2
GO:0051239	regulation of multicellular organismal process	0.662245	2
GO:0044264	cellular polysaccharide metabolic process	0.662245	4
GO:0006596	polyamine biosynthetic process	0.662245	1
GO:0006879	cellular iron ion homeostasis	0.662245	1
GO:0015977	carbon fixation	0.662245	1
GO:0019932	second-messenger-mediated signaling	0.662245	1
GO:0042214	terpene metabolic process	0.662245	1

GO:0046174	polyol catabolic process	0.662245	1
GO:0046365	monosaccharide catabolic process	0.662245	1
GO:0046417	chorismate metabolic process	0.662245	1
GO:0120252	hydrocarbon metabolic process	0.662245	1
GO:0044242	cellular lipid catabolic process	0.662245	2
GO:0016053	organic acid biosynthetic process	0.668713	7
GO:0046903	secretion	0.675929	2
GO:0009611	response to wounding	0.675929	1
GO:0009640	photomorphogenesis	0.675929	1
GO:0046164	alcohol catabolic process	0.675929	1
GO:0035966	response to topologically incorrect protein	0.675929	2
GO:0035967	cellular response to topologically incorrect protein	0.675929	2
GO:0061077	chaperone-mediated protein folding	0.675929	2
GO:0006575	cellular modified amino acid metabolic process	0.695031	3
GO:0006541	glutamine metabolic process	0.70051	1
GO:0009262	deoxyribonucleotide metabolic process	0.70051	1
GO:1901616	organic hydroxy compound catabolic process	0.70051	1
GO:0071554	cell wall organization or biogenesis	0.701862	6
GO:0006629	lipid metabolic process	0.71724	12
GO:0008283	cell population proliferation	0.71724	1
GO:0010228	vegetative to reproductive phase transition of meristem	0.71724	1
GO:0055072	iron ion homeostasis	0.71724	1
GO:0044282	small molecule catabolic process	0.71724	4
GO:0035556	intracellular signal transduction	0.71724	5
GO:0019752	carboxylic acid metabolic process	0.71724	12
GO:0009698	phenylpropanoid metabolic process	0.71724	2
GO:0006595	polyamine metabolic process	0.71724	1
GO:0009084	glutamine family amino acid biosynthetic process	0.71724	1
GO:0009909	regulation of flower development	0.71724	1
GO:0048831	regulation of shoot system development	0.71724	1
GO:0050821	protein stabilization	0.71724	1
GO:0097237	cellular response to toxic substance	0.71724	1
GO:1990748	cellular detoxification	0.71724	1
GO:0043436	oxoacid metabolic process	0.72019	12
GO:0006869	lipid transport	0.72019	2
GO:1990542	mitochondrial transmembrane transport	0.72019	2
GO:0006644	phospholipid metabolic process	0.72019	3
GO:0007018	microtubule-based movement	0.72019	1
GO:0022622	root system development	0.72019	1
GO:0031647	regulation of protein stability	0.72019	1
GO:0043650	dicarboxylic acid biosynthetic process	0.72019	1
GO:0048364	root development	0.72019	1
GO:0006066	alcohol metabolic process	0.725733	2
GO:0034220	ion transmembrane transport	0.725733	5

GO:0018105	peptidyl-serine phosphorylation	0.731856	2
GO:0046364	monosaccharide biosynthetic process	0.731856	1
GO:0046916	cellular transition metal ion homeostasis	0.731856	1
GO:0098754	detoxification	0.731856	1
GO:0010876	lipid localization	0.737537	2
GO:0018209	peptidyl-serine modification	0.737537	2
GO:0006090	pyruvate metabolic process	0.739002	2
GO:0006749	glutathione metabolic process	0.739002	2
GO:0006221	pyrimidine nucleotide biosynthetic process	0.739002	1
GO:0030244	cellulose biosynthetic process	0.739002	1
GO:0051274	beta-glucan biosynthetic process	0.739002	1
GO:0032268	regulation of cellular protein metabolic process	0.74283	5
GO:0006790	sulfur compound metabolic process	0.747117	5
GO:0006457	protein folding	0.747117	4
GO:0006885	regulation of pH	0.747117	1
GO:0048278	vesicle docking	0.747117	1
GO:0009308	amine metabolic process	0.747117	2
GO:0006082	organic acid metabolic process	0.747117	12
GO:0006520	cellular amino acid metabolic process	0.747117	6
GO:0050801	ion homeostasis	0.747117	2
GO:0055080	cation homeostasis	0.747117	2
GO:0098771	inorganic ion homeostasis	0.747117	2
GO:0000910	cytokinesis	0.747117	1
GO:0009627	systemic acquired resistance	0.747117	1
GO:0009767	photosynthetic electron transport chain	0.747117	1
GO:0055067	monovalent inorganic cation homeostasis	0.747117	1
GO:0051246	regulation of protein metabolic process	0.757391	5
GO:0006928	movement of cell or subcellular component	0.757391	1
GO:0009651	response to salt stress	0.757391	1
GO:0015698	inorganic anion transport	0.757391	1
GO:0055076	transition metal ion homeostasis	0.757391	1
GO:0000302	response to reactive oxygen species	0.776835	1
GO:0019748	secondary metabolic process	0.776835	2
GO:0006220	pyrimidine nucleotide metabolic process	0.7946	1
GO:0009605	response to external stimulus	0.7946	4
GO:0044272	sulfur compound biosynthetic process	0.7946	2
GO:0006563	L-serine metabolic process	0.7946	1
GO:0006970	response to osmotic stress	0.7946	1
GO:0008037	cell recognition	0.7946	1
GO:0009639	response to red or far red light	0.7946	1
GO:0040008	regulation of growth	0.7946	1
GO:0048544	recognition of pollen	0.7946	1
GO:0032502	developmental process	0.801255	9
GO:0009309	amine biosynthetic process	0.801255	1

GO:0042401	cellular biogenic amine biosynthetic process	0.801255	1
GO:0045047	protein targeting to ER	0.801255	1
GO:0072599	establishment of protein localization to endoplasmic reticulum	0.801255	1
GO:0009791	post-embryonic development	0.802083	3
GO:0022406	membrane docking	0.810154	1
GO:0072528	pyrimidine-containing compound biosynthetic process	0.810154	1
GO:0140056	organelle localization by membrane tethering	0.810154	1
GO:0009664	plant-type cell wall organization	0.823965	1
GO:0051651	maintenance of location in cell	0.823965	1
GO:0016051	carbohydrate biosynthetic process	0.823965	3
GO:0009073	aromatic amino acid family biosynthetic process	0.837811	1
GO:0071669	plant-type cell wall organization or biogenesis	0.844728	2
GO:0006906	vesicle fusion	0.844728	1
GO:2000241	regulation of reproductive process	0.844728	1
GO:0006839	mitochondrial transport	0.844728	2
GO:0006576	cellular biogenic amine metabolic process	0.844728	1
GO:0009875	pollen-pistil interaction	0.844728	1
GO:0042364	water-soluble vitamin biosynthetic process	0.844728	1
GO:0043086	negative regulation of catalytic activity	0.844728	1
GO:0044092	negative regulation of molecular function	0.844728	1
GO:0051235	maintenance of location	0.844728	1
GO:0006875	cellular metal ion homeostasis	0.850322	1
GO:0009070	serine family amino acid biosynthetic process	0.850322	1
GO:0009110	vitamin biosynthetic process	0.850322	1
GO:0044106	cellular amine metabolic process	0.850322	1
GO:0006081	cellular aldehyde metabolic process	0.855369	1
GO:0006887	exocytosis	0.855369	1
GO:0046434	organophosphate catabolic process	0.855369	1
GO:0090174	organelle membrane fusion	0.855369	1
GO:1901606	alpha-amino acid catabolic process	0.8817	1
GO:0098660	inorganic ion transmembrane transport	0.8817	2
GO:0009250	glucan biosynthetic process	0.899789	1
GO:0031146	SCF-dependent proteasomal ubiquitin-dependent protein catabolic process	0.899789	1
GO:0055065	metal ion homeostasis	0.899789	1
GO:0061025	membrane fusion	0.899789	1
GO:0048608	reproductive structure development	0.902234	2
GO:0061458	reproductive system development	0.902234	2
GO:0006767	water-soluble vitamin metabolic process	0.902234	1
GO:0009808	lignin metabolic process	0.902234	1
GO:0006766	vitamin metabolic process	0.90456	1
GO:0009069	serine family amino acid metabolic process	0.90456	1
GO:0048284	organelle fusion	0.90456	1
GO:0046034	ATP metabolic process	0.90456	2
GO:0009064	glutamine family amino acid metabolic process	0.911834	1

GO:0044093	positive regulation of molecular function	0.911834	1
GO:0009856	pollination	0.91603	1
GO:0044706	multi-multicellular organism process	0.91603	1
GO:0072527	pyrimidine-containing compound metabolic process	0.91603	1
GO:0032940	secretion by cell	0.925726	1
GO:0048731	system development	0.92837	3
GO:0040007	growth	0.932099	1
GO:0006873	cellular ion homeostasis	0.937891	1
GO:0030003	cellular cation homeostasis	0.937891	1
GO:0044255	cellular lipid metabolic process	0.937891	6
GO:0044283	small molecule biosynthetic process	0.937891	7
GO:0009908	flower development	0.937891	1
GO:0090567	reproductive shoot system development	0.937891	1
GO:0006612	protein targeting to membrane	0.943218	1
GO:0009063	cellular amino acid catabolic process	0.943218	1
GO:0140352	export from cell	0.95115	1
GO:0048878	chemical homeostasis	0.958903	2
GO:0016125	sterol metabolic process	0.971594	1
GO:0099402	plant organ development	0.971594	1
GO:0003006	developmental process involved in reproduction	0.971594	2
GO:0070972	protein localization to endoplasmic reticulum	0.986477	1
GO:0055082	cellular chemical homeostasis	0.987539	1
GO:0006650	glycerophospholipid metabolic process	0.987539	1
GO:0009832	plant-type cell wall biogenesis	0.987539	1
GO:0019637	organophosphate metabolic process	0.987539	6
GO:0009117	nucleotide metabolic process	0.987539	3
GO:0065002	intracellular protein transmembrane transport	0.987539	1
GO:0008654	phospholipid biosynthetic process	0.987539	1
GO:0043648	dicarboxylic acid metabolic process	0.987539	1
GO:0045893	positive regulation of transcription, DNA-templated	0.987539	3
GO:1902680	positive regulation of RNA biosynthetic process	0.987539	3
GO:1903508	positive regulation of nucleic acid-templated transcription	0.987539	3
GO:0070646	protein modification by small protein removal	0.987539	1
GO:0071806	protein transmembrane transport	0.987539	1
GO:0007275	multicellular organism development	0.987539	4
GO:0033692	cellular polysaccharide biosynthetic process	0.987539	1
GO:0032501	multicellular organismal process	0.987539	5
GO:0016050	vesicle organization	0.987539	1
GO:0022900	electron transport chain	0.987539	1
GO:0051704	multi-organism process	0.987539	1
GO:0006753	nucleoside phosphate metabolic process	0.987539	3
GO:0008202	steroid metabolic process	0.987539	1
GO:0000003	reproduction	0.987539	3
GO:0022414	reproductive process	0.987539	3

GO:0051254	positive regulation of RNA metabolic process	0.987539	3
GO:0046486	glycerolipid metabolic process	0.987539	1
GO:0009150	purine ribonucleotide metabolic process	0.995336	2
GO:0045935	positive regulation of nucleobase-containing compound metabolic process	0.998459	3
GO:0006163	purine nucleotide metabolic process	0.999923	2
GO:0000271	polysaccharide biosynthetic process	0.999923	1
GO:0061024	membrane organization	0.999923	2
GO:0046395	carboxylic acid catabolic process	0.999923	1
GO:0051640	organelle localization	0.999923	1
GO:0098662	inorganic cation transmembrane transport	0.999923	1
GO:0009259	ribonucleotide metabolic process	0.999923	2
GO:0045944	positive regulation of transcription by RNA polymerase II	0.999923	1
GO:0009891	positive regulation of biosynthetic process	0.999923	3
GO:0010557	positive regulation of macromolecule biosynthetic process	0.999923	3
GO:0031328	positive regulation of cellular biosynthetic process	0.999923	3
GO:0034637	cellular carbohydrate biosynthetic process	0.999923	1
GO:0072521	purine-containing compound metabolic process	0.999923	2
GO:0016054	organic acid catabolic process	0.999923	1
GO:0042592	homeostatic process	0.999923	2
GO:0098655	cation transmembrane transport	0.999923	1
GO:0019693	ribose phosphate metabolic process	0.999923	2
GO:0090150	establishment of protein localization to membrane	0.999923	1
GO:0016310	phosphorylation	0.999923	5
GO:1903047	mitotic cell cycle process	0.999923	1
GO:0033554	cellular response to stress	0.999923	4
GO:0048583	regulation of response to stimulus	0.999923	1
GO:0019725	cellular homeostasis	0.999923	1
GO:0006357	regulation of transcription by RNA polymerase II	0.999923	3
GO:0006486	protein glycosylation	0.999923	1
GO:0009101	glycoprotein biosynthetic process	0.999923	1
GO:0043413	macromolecule glycosylation	0.999923	1
GO:0051301	cell division	0.999923	1
GO:0070085	glycosylation	0.999923	1
GO:0072330	monocarboxylic acid biosynthetic process	0.999923	1
GO:0006468	protein phosphorylation	0.999923	3
GO:0048367	shoot system development	0.999923	1
GO:0009100	glycoprotein metabolic process	0.999923	1
GO:0002181	cytoplasmic translation	0.999923	1
GO:0018193	peptidyl-amino acid modification	0.999923	2
GO:0009165	nucleotide biosynthetic process	0.999923	1
GO:1901293	nucleoside phosphate biosynthetic process	0.999923	1
GO:0009057	macromolecule catabolic process	0.999923	8
GO:0072657	protein localization to membrane	0.999923	1
GO:0048856	anatomical structure development	0.999923	4

GO:0006366	transcription by RNA polymerase II	0.999923	3
GO:0055086	nucleobase-containing small molecule metabolic process	0.999923	3
GO:0042546	cell wall biogenesis	0.999923	1
GO:0090407	organophosphate biosynthetic process	0.999923	2
GO:0051173	positive regulation of nitrogen compound metabolic process	0.999923	3
GO:0010604	positive regulation of macromolecule metabolic process	0.999923	3
GO:0031325	positive regulation of cellular metabolic process	0.999923	3
GO:0009893	positive regulation of metabolic process	0.999923	3
GO:0007017	microtubule-based process	0.999923	1
GO:0009890	negative regulation of biosynthetic process	0.999923	1
GO:0008610	lipid biosynthetic process	0.999923	3
GO:0006812	cation transport	0.999923	1
GO:0000278	mitotic cell cycle	0.999923	1
GO:0031324	negative regulation of cellular metabolic process	0.999923	1
GO:0048522	positive regulation of cellular process	0.999923	3
GO:0019439	aromatic compound catabolic process	0.999923	1
GO:0022402	cell cycle process	0.999923	1
GO:1901361	organic cyclic compound catabolic process	0.999923	1
GO:0006605	protein targeting	0.999923	1
GO:0065008	regulation of biological quality	0.999923	3
GO:1901137	carbohydrate derivative biosynthetic process	0.999923	2
GO:0048518	positive regulation of biological process	0.999923	3
GO:0072594	establishment of protein localization to organelle	0.999923	1
GO:1901135	carbohydrate derivative metabolic process	0.999923	4
GO:0043161	proteasome-mediated ubiquitin-dependent protein catabolic process	0.999923	1
GO:0048523	negative regulation of cellular process	0.999923	1
GO:0010498	proteasomal protein catabolic process	0.999923	1
GO:0009892	negative regulation of metabolic process	0.999923	1
GO:0033365	protein localization to organelle	0.999923	1
GO:0070647	protein modification by small protein conjugation or removal	0.999923	2
GO:0016567	protein ubiquitination	0.999923	1
GO:0007049	cell cycle	0.999923	1
GO:0032446	protein modification by small protein conjugation	0.999923	1
GO:0048519	negative regulation of biological process	0.999923	1
GO:0046907	intracellular transport	0.999923	3
GO:0006886	intracellular protein transport	0.999923	2
GO:0051649	establishment of localization in cell	0.999923	3
GO:0006511	ubiquitin-dependent protein catabolic process	0.999923	1
GO:0019941	modification-dependent protein catabolic process	0.999923	1
GO:0043632	modification-dependent macromolecule catabolic process	0.999923	1
GO:0042886	amide transport	0.999923	3
GO:0016192	vesicle-mediated transport	0.999923	1
GO:0065003	protein-containing complex assembly	0.999923	1
GO:0034613	cellular protein localization	0.999923	2

GO:0070727	cellular macromolecule localization	0.999923	2
GO:0006508	proteolysis	0.999923	2
GO:0022607	cellular component assembly	0.999923	2
GO:0044257	cellular protein catabolic process	0.999923	1
GO:0051603	proteolysis involved in cellular protein catabolic process	0.999923	1
GO:0030163	protein catabolic process	0.999923	1
GO:1901565	organonitrogen compound catabolic process	0.999923	2
GO:0043933	protein-containing complex subunit organization	0.999923	1
GO:0015031	protein transport	0.999923	2
GO:0045184	establishment of protein localization	0.999923	2
GO:0015833	peptide transport	0.999923	2
GO:0006396	RNA processing	0.999923	1
GO:0044265	cellular macromolecule catabolic process	0.999923	1
GO:0006412	translation	0.999923	2
GO:0043043	peptide biosynthetic process	0.999923	2
GO:0005576	extracellular region	5.55E-05	24
GO:0005911	cell–cell junction	7.91E-05	11
GO:0009506	plasmodesma	7.91E-05	11
GO:0030054	cell junction	7.91E-05	11
GO:0055044	symplast	7.91E-05	11
GO:0070161	anchoring junction	7.91E-05	11
GO:0009522	photosystem I	0.001061	6
GO:0009505	plant-type cell wall	0.003628	6
GO:0010287	plastoglobule	0.019016	3
GO:0005618	cell wall	0.035387	11
GO:0009521	photosystem	0.035387	6
GO:0030312	external encapsulating structure	0.040714	11
GO:0009523	photosystem II	0.043618	5
GO:0009535	chloroplast thylakoid membrane	0.075652	8
GO:0055035	plastid thylakoid membrane	0.075652	8
GO:0042651	thylakoid membrane	0.132579	8
GO:0034357	photosynthetic membrane	0.140468	8
GO:0009534	chloroplast thylakoid	0.140901	8
GO:0031976	plastid thylakoid	0.140901	8
GO:0031304	intrinsic component of mitochondrial inner membrane	0.260928	2
GO:0031305	integral component of mitochondrial inner membrane	0.260928	2
GO:0005887	integral component of plasma membrane	0.260928	3
GO:0009579	thylakoid	0.260928	8
GO:0005802	trans-Golgi network	0.358362	3
GO:0032592	integral component of mitochondrial membrane	0.358362	2
GO:0098573	intrinsic component of mitochondrial membrane	0.358362	2
GO:0009570	chloroplast stroma	0.515867	4
GO:0009532	plastid stroma	0.545684	4
GO:0005791	rough endoplasmic reticulum	0.571721	1

GO:0030867	rough endoplasmic reticulum membrane	0.571721	1
GO:0031226	intrinsic component of plasma membrane	0.583944	4
GO:0005615	extracellular space	0.654868	2
GO:0030687	preribosome, large subunit precursor	0.681671	1
GO:0009941	chloroplast envelope	0.857113	3
GO:0005774	vacuolar membrane	0.857113	3
GO:0009526	plastid envelope	0.885724	3
GO:0009705	plant-type vacuole membrane	0.892902	1
GO:0005875	microtubule associated complex	0.924437	1
GO:0000325	plant-type vacuole	0.999785	1
GO:0031201	SNARE complex	0.999785	1
GO:0019005	SCF ubiquitin ligase complex	0.999785	1
GO:0031225	anchored component of membrane	0.999785	2
GO:0098796	membrane protein complex	0.999785	8
GO:0098791	Golgi apparatus subcompartment	0.999785	4
GO:0030684	preribosome	0.999785	1
GO:0031301	integral component of organelle membrane	0.999785	2
GO:0031300	intrinsic component of organelle membrane	0.999785	2
GO:0005773	vacuole	0.999785	3
GO:0005768	endosome	0.999785	2
GO:0031461	cullin-RING ubiquitin ligase complex	0.999785	1
GO:0046658	anchored component of plasma membrane	0.999785	1
GO:0031967	organelle envelope	0.999785	5
GO:0031975	envelope	0.999785	5
GO:0140534	endoplasmic reticulum protein-containing complex	0.999785	1
GO:0005743	mitochondrial inner membrane	0.999785	2
GO:0022627	cytosolic small ribosomal subunit	0.999785	1
GO:0005874	microtubule	0.999785	1
GO:0005794	Golgi apparatus	0.999785	6
GO:0099081	supramolecular polymer	0.999785	1
GO:0099512	supramolecular fiber	0.999785	1
GO:0099513	polymeric cytoskeletal fiber	0.999785	1
GO:0000151	ubiquitin ligase complex	0.999785	1
GO:0019866	organelle inner membrane	0.999785	2
GO:0015630	microtubule cytoskeleton	0.999785	1
GO:0098588	bounding membrane of organelle	0.999785	5
GO:0031966	mitochondrial membrane	0.999785	2
GO:0015935	small ribosomal subunit	0.999785	1
GO:0005740	mitochondrial envelope	0.999785	2
GO:0048046	apoplast	0.999785	1
GO:0031410	cytoplasmic vesicle	0.999785	2
GO:0097708	intracellular vesicle	0.999785	2
GO:0031982	vesicle	0.999785	2
GO:0099080	supramolecular complex	0.999785	1

GO:0000139	Golgi membrane	0.999785	1
GO:0005856	cytoskeleton	0.999785	1
GO:0005789	endoplasmic reticulum membrane	0.999785	1
GO:0042175	nuclear outer membrane-endoplasmic reticulum membrane network	0.999785	1
GO:0098827	endoplasmic reticulum subcompartment	0.999785	1
GO:0022626	cytosolic ribosome	0.999785	1
GO:1990234	transferase complex	0.999785	1
GO:0044391	ribosomal subunit	0.999785	1
GO:0005739	mitochondrion	0.999785	3
GO:0140535	intracellular protein-containing complex	0.999785	1
GO:0005840	ribosome	0.999785	1
GO:0005783	endoplasmic reticulum	0.999785	1
GO:0046906	tetrapyrrole binding	7.24E-06	24
GO:0004601	peroxidase activity	0.000156	12
GO:0016684	oxidoreductase activity, acting on peroxide as acceptor	0.000156	12
GO:0020037	heme binding	0.000369	19
GO:0016209	antioxidant activity	0.001024	12
GO:0004857	enzyme inhibitor activity	0.002368	13
GO:0008171	O-methyltransferase activity	0.002604	6
GO:0008168	methyltransferase activity	0.007279	12
GO:0016168	chlorophyll binding	0.00819	5
GO:0033293	monocarboxylic acid binding	0.009578	6
GO:0016741	transferase activity, transferring one-carbon groups	0.010364	12
GO:0010427	abscisic acid binding	0.0174	5
GO:0019840	isoprenoid binding	0.02476	5
GO:0042562	hormone binding	0.02618	5
GO:0004864	protein phosphatase inhibitor activity	0.02618	5
GO:0019212	phosphatase inhibitor activity	0.02618	5
GO:0043178	alcohol binding	0.035429	5
GO:0031406	carboxylic acid binding	0.041773	6
GO:0008810	cellulase activity	0.041773	3
GO:0008757	S-adenosylmethionine-dependent methyltransferase activity	0.041773	7
GO:0080030	methyl indole-3-acetate esterase activity	0.044994	3
GO:0080031	methyl salicylate esterase activity	0.044994	3
GO:0080032	methyl jasmonate esterase activity	0.044994	3
GO:0052689	carboxylic ester hydrolase activity	0.04858	8
GO:0043177	organic acid binding	0.083849	5
GO:0019208	phosphatase regulator activity	0.084035	5
GO:0019888	protein phosphatase regulator activity	0.084035	5
GO:0004659	prenyltransferase activity	0.103741	3
GO:0030234	enzyme regulator activity	0.159644	13
GO:0038023	signaling receptor activity	0.189153	5
GO:0016765	transferase activity, transferring alkyl or aryl (other than methyl) groups	0.189153	6
GO:0120014	phospholipid transfer activity	0.209648	2

GO:0005548	phospholipid transporter activity	0.210605	2
GO:0120013	lipid transfer activity	0.210605	2
GO:0008289	lipid binding	0.210605	8
GO:0004866	endopeptidase inhibitor activity	0.210605	3
GO:0008483	transaminase activity	0.210605	3
GO:0016769	transferase activity, transferring nitrogenous groups	0.210605	3
GO:0030414	peptidase inhibitor activity	0.210605	3
GO:0061134	peptidase regulator activity	0.210605	3
GO:0061135	endopeptidase regulator activity	0.210605	3
GO:0015293	symporter activity	0.212414	4
GO:0043531	ADP binding	0.212414	4
GO:0098772	molecular function regulator	0.220338	14
GO:0060089	molecular transducer activity	0.226465	5
GO:0004497	monooxygenase activity	0.274201	8
GO:0031072	heat shock protein binding	0.290234	3
GO:0046910	pectinesterase inhibitor activity	0.303564	3
GO:0022804	active transmembrane transporter activity	0.303564	9
GO:0045330	aspartyl esterase activity	0.351641	3
GO:0015399	primary active transmembrane transporter activity	0.365442	3
GO:0044183	protein folding chaperone	0.378251	2
GO:0051787	misfolded protein binding	0.378251	2
GO:0003993	acid phosphatase activity	0.403838	2
GO:0030599	pectinesterase activity	0.441878	3
GO:0051213	dioxygenase activity	0.506659	4
GO:0016298	lipase activity	0.506659	3
GO:0005506	iron ion binding	0.506659	8
GO:0004553	hydrolase activity, hydrolyzing O-glycosyl compounds	0.520938	7
GO:0016705	oxidoreductase activity, acting on paired donors, with incorporation or reduction of molecular oxygen	0.530291	8
GO:0005342	organic acid transmembrane transporter activity	0.534686	2
GO:0046943	carboxylic acid transmembrane transporter activity	0.534686	2
GO:0004620	phospholipase activity	0.557857	2
GO:0000976	transcription regulatory region sequence-specific DNA binding	0.563064	8
GO:0001067	regulatory region nucleic acid binding	0.563064	8
GO:1904680	peptide transmembrane transporter activity	0.563064	2
GO:0005319	lipid transporter activity	0.585073	2
GO:1990837	sequence-specific double-stranded DNA binding	0.587054	8
GO:0051082	unfolded protein binding	0.587054	4
GO:0005516	calmodulin binding	0.587054	3
GO:0042626	ATPase-coupled transmembrane transporter activity	0.587054	2
GO:0042887	amide transmembrane transporter activity	0.587054	2
GO:0043565	sequence-specific DNA binding	0.587054	14
GO:0004683	calmodulin-dependent protein kinase activity	0.587054	2
GO:0008514	organic anion transmembrane transporter activity	0.587054	2

GO:0009931	calcium-dependent protein serine/threonine kinase activity	0.587054	2
GO:0010857	calcium-dependent protein kinase activity	0.587054	2
GO:0015291	secondary active transmembrane transporter activity	0.587054	6
GO:0016160	amylase activity	0.587054	1
GO:0022843	voltage-gated cation channel activity	0.587054	1
GO:0030545	receptor regulator activity	0.587054	1
GO:0030546	signaling receptor activator activity	0.587054	1
GO:0043621	protein self-association	0.587054	1
GO:0048018	receptor ligand activity	0.587054	1
GO:0051879	Hsp90 protein binding	0.587054	1
GO:0072341	modified amino acid binding	0.587054	1
GO:0004364	glutathione transferase activity	0.587054	2
GO:0016701	oxidoreductase activity, acting on single donors with incorporation of molecular oxygen	0.587054	2
GO:0008194	UDP-glycosyltransferase activity	0.587054	6
GO:0047213	anthocyanidin 3-O-glucosyltransferase activity	0.587054	3
GO:0016798	hydrolase activity, acting on glycosyl bonds	0.587054	7
GO:0008146	sulfotransferase activity	0.587054	1
GO:0016832	aldehyde-lyase activity	0.587054	1
GO:0047372	acylglycerol lipase activity	0.587054	1
GO:0035251	UDP-glucosyltransferase activity	0.587054	4
GO:0005507	copper ion binding	0.587054	3
GO:0050660	flavin adenine dinucleotide binding	0.587054	3
GO:0050661	NADP binding	0.587054	2
GO:0071949	FAD binding	0.587054	2
GO:0000030	mannosyltransferase activity	0.588037	1
GO:0015923	mannosidase activity	0.588037	1
GO:0016638	oxidoreductase activity, acting on the CH-NH2 group of donors	0.588037	1
GO:0005267	potassium channel activity	0.594542	1
GO:0008378	galactosyltransferase activity	0.594542	1
GO:0016759	cellulose synthase activity	0.594542	1
GO:0016760	cellulose synthase (UDP-forming) activity	0.594542	1
GO:0016846	carbon-sulfur lyase activity	0.594542	1
GO:0050145	nucleoside monophosphate kinase activity	0.594542	1
GO:0008081	phosphoric diester hydrolase activity	0.626941	1
GO:0005543	phospholipid binding	0.644565	2
GO:0005384	manganese ion transmembrane transporter activity	0.646074	1
GO:0047834	D-threo-aldose 1-dehydrogenase activity	0.646074	1
GO:0003690	double-stranded DNA binding	0.6499	8
GO:0005509	calcium ion binding	0.674061	6
GO:0016887	ATPase activity	0.701307	9
GO:0016709	oxidoreductase activity, acting on paired donors, with incorporation or reduction of molecular oxygen, NAD(P)H as one donor, and incorporation of one atom of oxygen	0.701307	2

GO:0003777	microtubule motor activity	0.701307	1
GO:0004568	chitinase activity	0.701307	1
GO:1990939	ATP-dependent microtubule motor activity	0.701307	1
GO:0008509	anion transmembrane transporter activity	0.701307	5
GO:0004190	aspartic-type endopeptidase activity	0.701307	2
GO:0070001	aspartic-type peptidase activity	0.701307	2
GO:0016776	phosphotransferase activity, phosphate group as acceptor	0.702641	1
GO:0016782	transferase activity, transferring sulfur-containing groups	0.702641	1
GO:0022853	active ion transmembrane transporter activity	0.702641	2
GO:0005102	signaling receptor binding	0.702641	1
GO:0016840	carbon-nitrogen lyase activity	0.702641	1
GO:0048038	quinone binding	0.702641	1
GO:0046527	glucosyltransferase activity	0.702641	4
GO:0030246	carbohydrate binding	0.702641	3
GO:0046983	protein dimerization activity	0.702641	7
GO:0003680	minor groove of adenine-thymine-rich DNA binding	0.702641	1
GO:0005244	voltage-gated ion channel activity	0.702641	1
GO:0008320	protein transmembrane transporter activity	0.702641	1
GO:0015299	solute:proton antiporter activity	0.702641	1
GO:0022832	voltage-gated channel activity	0.702641	1
GO:0022884	macromolecule transmembrane transporter activity	0.702641	1
GO:0140318	protein transporter activity	0.702641	1
GO:1901681	sulfur compound binding	0.702641	1
GO:0016616	oxidoreductase activity, acting on the CH-OH group of donors, NAD or NADP as acceptor	0.702641	4
GO:0003774	motor activity	0.712395	1
GO:0016628	oxidoreductase activity, acting on the CH-CH group of donors, NAD or NADP as acceptor	0.712395	1
GO:0015318	inorganic molecular entity transmembrane transporter activity	0.714864	8
GO:0000217	DNA secondary structure binding	0.714864	1
GO:0015298	solute:cation antiporter activity	0.714864	1
GO:0016857	racemase and epimerase activity, acting on carbohydrates and derivatives	0.714864	1
GO:0016788	hydrolase activity, acting on ester bonds	0.733159	13
GO:0019205	nucleobase-containing compound kinase activity	0.736921	1
GO:0016614	oxidoreductase activity, acting on CH-OH group of donors	0.736921	4
GO:0015267	channel activity	0.736921	3
GO:0022803	passive transmembrane transporter activity	0.736921	3
GO:0005484	SNAP receptor activity	0.736921	1
GO:0016702	oxidoreductase activity, acting on single donors with incorporation of molecular oxygen, incorporation of two atoms of oxygen	0.736921	1
GO:0051539	4 iron, 4 sulfur cluster binding	0.736921	2
GO:0005372	water transmembrane transporter activity	0.736921	1
GO:0015250	water channel activity	0.736921	1
GO:0080043	quercetin 3-O-glucosyltransferase activity	0.736921	1

GO:0080044	quercetin 7-O-glucosyltransferase activity	0.736921	1
GO:0015075	ion transmembrane transporter activity	0.753384	9
GO:0033218	amide binding	0.757923	2
GO:0015079	potassium ion transmembrane transporter activity	0.757923	1
GO:0016620	oxidoreductase activity, acting on the aldehyde or oxo group of donors, NAD or NADP as acceptor	0.763012	1
GO:0016854	racemase and epimerase activity	0.763012	1
GO:0004222	metalloendopeptidase activity	0.763012	1
GO:0022836	gated channel activity	0.763012	1
GO:0015103	inorganic anion transmembrane transporter activity	0.763012	1
GO:0019829	ATPase-coupled cation transmembrane transporter activity	0.763012	1
GO:0042625	ATPase-coupled ion transmembrane transporter activity	0.763012	1
GO:0046915	transition metal ion transmembrane transporter activity	0.763012	1
GO:0030170	pyridoxal phosphate binding	0.763012	2
GO:0070279	vitamin B6 binding	0.763012	2
GO:0016757	transferase activity, transferring glycosyl groups	0.801866	8
GO:0000149	SNARE binding	0.801866	1
GO:0140104	molecular carrier activity	0.801866	1
GO:0016879	ligase activity, forming carbon-nitrogen bonds	0.810185	1
GO:0016758	transferase activity, transferring hexosyl groups	0.816375	5
GO:0106310	protein serine kinase activity	0.816375	2
GO:0106311	protein threonine kinase activity	0.816375	2
GO:0030145	manganese ion binding	0.816375	1
GO:0008270	zinc ion binding	0.8165	7
GO:0030674	protein-macromolecule adaptor activity	0.818921	1
GO:0016903	oxidoreductase activity, acting on the aldehyde or oxo group of donors	0.821246	1
GO:0046873	metal ion transmembrane transporter activity	0.821246	2
GO:0005261	cation channel activity	0.823012	1
GO:0060090	molecular adaptor activity	0.823012	1
GO:0019842	vitamin binding	0.829295	2
GO:0016627	oxidoreductase activity, acting on the CH-CH group of donors	0.829295	1
GO:0022890	inorganic cation transmembrane transporter activity	0.829295	4
GO:0016829	lyase activity	0.829295	4
GO:0004674	protein serine/threonine kinase activity	0.829295	7
GO:0015297	antiporter activity	0.866301	2
GO:0016836	hydro-lyase activity	0.866301	1
GO:0008237	metallopeptidase activity	0.866301	1
GO:0008324	cation transmembrane transporter activity	0.866301	4
GO:0008017	microtubule binding	0.866301	1
GO:0000978	RNA polymerase II cis-regulatory region sequence-specific DNA binding	0.866301	2
GO:0000987	cis-regulatory region sequence-specific DNA binding	0.866301	2
GO:0051536	iron-sulfur cluster binding	0.866301	2
GO:0051540	metal cluster binding	0.866301	2
GO:0042578	phosphoric ester hydrolase activity	0.868037	4

GO:0003743	translation initiation factor activity	0.869004	1
GO:0004386	helicase activity	0.869004	1
GO:0015631	tubulin binding	0.869004	1
GO:0042277	peptide binding	0.869004	1
GO:0051287	NAD binding	0.869004	1
GO:0016830	carbon-carbon lyase activity	0.871082	1
GO:0004842	ubiquitin-protein transferase activity	0.890714	4
GO:0005216	ion channel activity	0.891636	1
GO:0000977	RNA polymerase II transcription regulatory region sequence-specific DNA binding	0.896776	2
GO:0019787	ubiquitin-like protein transferase activity	0.900449	4
GO:0017111	nucleoside-triphosphatase activity	0.900449	9
GO:0016651	oxidoreductase activity, acting on NAD(P)H	0.904467	1
GO:0016791	phosphatase activity	0.919085	3
GO:0004175	endopeptidase activity	0.935225	3
GO:0016835	carbon-oxygen lyase activity	0.93857	1
GO:0015078	proton transmembrane transporter activity	0.944799	1
GO:0000981	DNA-binding transcription factor activity, RNA polymerase II-specific	0.944799	2
GO:0008135	translation factor activity, RNA binding	0.945008	1
GO:0061630	ubiquitin protein ligase activity	0.945008	1
GO:0003729	mRNA binding	0.945008	2
GO:0061659	ubiquitin-like protein ligase activity	0.945008	1
GO:0090079	translation regulator activity, nucleic acid binding	0.945008	1
GO:0045182	translation regulator activity	0.945008	1
GO:0008092	cytoskeletal protein binding	0.952971	1
GO:0008233	peptidase activity	0.962247	4
GO:0016874	ligase activity	0.962421	1
GO:0009055	electron transfer activity	0.976632	1
GO:0016853	isomerase activity	0.985786	1
GO:0003735	structural constituent of ribosome	0.99998	1
GO:0005198	structural molecule activity	0.99998	1

ID: GO terms number. corrected p value. Count: the number of genes enriched in each GO term.
